# Assessment of microbial contamination in laser materials processing laboratories used for prototyping of biomedical devices

**DOI:** 10.1099/acmi.0.000494.v3

**Published:** 2023-12-12

**Authors:** Yinka M. Somorin, Gerard M. O'Connor

**Affiliations:** ^1^​ National Centre for Laser Applications (NCLA), School of Natural Sciences, University of Galway, Galway, Ireland; ^2^​ Irish Photonic Integration Centre (IPIC), Tyndall National Institute, Cork, Ireland

**Keywords:** bioburden, clean room, laser, medical devices, microbial contamination, particle count, pilot line

## Abstract

Microbial contamination of medical devices during pilot production can be a significant barrier as the laboratory environment is a source of contamination. There is limited information on microbial contaminants in laser laboratories and environments involved in the pilot production of medical devices. This study aimed to determine the bioburden and microbial contaminants present in three laser laboratories – an ISO class 7 clean room, a pilot line facility and a standard laser laboratory. Microbiological air sampling was by passive air sampling using settle plates and the identity of isolates was confirmed by DNA sequencing. Particulate matter was analysed using a portable optical particle counter. Twenty bacterial and 16 fungal genera were isolated, with the genera *Staphylococcus* and *Micrococcus* being predominant. Most isolates are associated with skin, mouth, or upper respiratory tract. There was no significant correlation between microbial count and PM_2.5_ concentration in the three laboratories. There were low levels but diverse microbial population in the laser-processing environments. Pathogenic bacteria such as *Acinetobacter baumannii* and *Candida parapsilosis* were isolated in those environments. These results provide data that will be useful for developing a contamination control plan for controlling microbial contamination and facilitating advanced manufacturing of laser-based pilot production of medical devices.

## Data Summary

The authors confirm all supporting data have been provided within the article.

## Introduction

Laser technologies are an important prototyping tool for modifying materials and imbuing them with new properties. Laser technologies are applied in manufacturing various medical devices. Laser cutting has found wide application in manufacturing flexible implantable devices such as stents, catheters, meshes and orthodontic wires [1, 2]. Furthermore, laser micromachining has enabled high-precision, non-destructive structuring used for producing delicate features on different metal and polymer surfaces using ultrashort pulse lasers. It has been applied in fabricating microneedle drug delivery systems, drug infusion catheters, coronary stents and micro-electrodes [3, 4].

Laser structuring often imparts unique properties to materials for potential biomedical applications. For example, using melt-free ultra-short laser femtosecond pulses, our research group have been able to selectively enhance the crystalline structure in molybdenum thin films [5], and improve the conductivity and electrical properties of thin film materials [6–8], which could have application in signal enhancement for sensor development. Further, femto- and picosecond lasers have also been used to generate nano-topographical structures on platinum iridium (Pt/Ir) microelectrode probes, leading to improved electrochemical properties and stimulation of key neural functions [9].

Contamination of medical devices by micro-organisms compromises the quality of such devices, hence the requirement to assess the risk of contaminants, including monitoring the manufacturing environment and the manufacturing process, to eliminate or reduce the possibility of device-associated infections in animal trials and during subsequent use in humans [10]. Microbial contamination of medical devices can arise from raw materials [11], personnel or the manufacturing environment [12]. Although terminal sterilization is used for decontaminating medical devices, some terminal sterilization approaches, such as gamma irradiation and ethylene oxide, have limited applications in tissue engineering and regenerative medicine applications due to material incompatibility [13]. Hence, the emphasis on contamination control during medical device manufacturing.

While there are efforts to reduce microbial contamination of medical devices, such as redesigning packages and modifying their opening techniques for aseptic transfer [14], the reduction of contamination during production remains a priority. Since the environment is a major source of microbial contamination during manufacturing, and little is known about the levels and types of microbial contaminants in environments where lasers are used to process materials for biomedical prototypes, the following questions are raised. (1) What are the micro-organisms present in typical laser laboratories, which have laser sources, beam delivery systems, motion stages and air-handling systems? (2) What are the levels of these micro-organisms? (3) Are there similarities in the levels and types of micro-organisms in the different laser laboratories? Hence, the aim of this study was to determine the level of microbial contamination, types of microbial contaminants present and whether there are similarities or differences in the microbial contamination in three different laser materials processing laboratories. The results support the development of a contamination control plan for manufacturing laser-enabled regenerative medical products.

## Methods

### Study location

This study was conducted in three laser laboratories, namely the ISO class 7 clean room, a newly refurbished pilot line facility and a standard laser laboratory. The clean room houses a femtosecond laser system, has an H14 high-efficiency particulate air (HEPA)-filtered air exchange system and requires the use of shoe covers, laboratory coat and face coverings as personal protective equipment. The pilot line facility is a newly refurbished laboratory that houses a new multifunctional manufacturing line for pilot production of medical devices. The pilot manufacturing line comprises three separate workstations. Workstation 1 is an ultraviolet laser ablation workstation, workstation 2 is an infrared laser sintering/ablation workstation and workstation 3 is a micro-droplet printing workstation. The pilot line facility requires the use of laboratory coat and face covering. The standard laser laboratory houses a CO_2_ laser system and only requires the use of a face covering. Air handling in the pilot line and the standard laser laboratories is controlled by a Mitsubishi Electric CITY MULTI system and consists of 30–40 % air from outside with recycled air from the laboratory. All laboratories have local air extraction from the laser material interaction zone. The laboratories had low occupancy rates, with between one and three individuals in the room while it was being sampled during this study. Further descriptions of the laboratory environment are presented in Table 1.

**Table 1.** Description of the laser materials processing environments

	Clean room	Pilot line facility	Standard laser laboratory
Free space/volume of the laboratories	125 m^3^	200 m^3^	65 m^3^
Pressure	Positive	Neutral	
Source of the air intake	30–40 % outside air+recycled air from the laboratory filtered through H14 HEPA filter	Reclaimed laboratory air mixed with 30–50 % fresh air from outside building. Previously believed to result in approx. six air changes h^−1^. Shared air conditioning system but no filtering	
Frequency of air exchanges	32 changes h^−1^	na	
Inner sealing provided by walls/ floor surfaces of the laboratories?	Polyester-coated insulated panels to form walls/partitions and arranged to allow through cavity air flow	PVC-based easywipe walls	Painted wood panel with smooth finish
Room occupancy (during sampling period)	1–2	1–3	0–1
Clothing PPE	Laboratory coat (gowning in a separate, enclosed space) Shoe cover/dedicated shoes Face coverings (worn during sampling period because of COVID-19 regulations)	Laboratory coat (worn in the same space) Face coverings (worn during sampling period because of COVID-19 regulations)	Face coverings (worn during sampling period because of COVID-19 regulations)

### Microbial air sampling and colony count

The microbiological air sampling was done by passive air sampling using settle plates and was monitored for 5 consecutive weeks September–October 2021. Tryptone soy agar (TSA) and malt extract agar (MEA) plates were used for bacterial and fungal counts, respectively. During sampling, four TSA and MEA plates each were opened and exposed to the air at pre-defined positions in each of the rooms and within the pilot line workstations for 1 h. Each location was divided into four spots to ensure the full coverage of the location. After exposure, the plates were covered, labelled and taken to the incubator. TSA plates were incubated at 37 °C for 48 h and MEA plates were incubated at 30 °C for 5 days. The number of colonies in each plate was counted and recorded and the median colony number for each location determined. The limit of detection (LOD) is 1 c.f.u./plate.

### Identification of micro-organisms

Bacterial and fungal isolates were subcultured on TSA and MEA, respectively, and their morphological characteristics recorded. Identification of the isolates was confirmed using DNA-based approaches. 16S rDNA gene sequencing was used for bacterial identification using primers 27F (5′-AGAGTTTGATCMTGGCTCAG-3′) and 1492R (5′-TACGGYTACCTTGTTACGACTT-3′) [15], while fungi were identified by sequencing the ITS region and large subunit ribosomal RNA genes using the ITS1F (5′-CTTGGTCATTTAGAGGAAGTAA-3′) and LR3 (5′-CCGTGTTTCAAGACGGG-3′) [16]. For bacteria, DNA template was obtained using single colonies resuspended in 250 µl of sterile nuclease-free water. Fungal DNA was extracted using a DNeasy blood and tissue kit (Qiagen) according to the manufacturer’s instructions. One microlitre of extracted DNA/colony suspension (DNA template) was used in the PCR using Phusion High–Fidelity DNA Polymerase (Thermo Scientific Fisher, USA). PCR products were purified using a QIAquick Gel Extraction kit (Qiagen) and sequencing was performed by Eurofins Genomics (UK). Sequences were assembled on DNA Baser version 5 (Heracle BioSoft, USA) and the identity of the sequences confirmed by basic local alignment search tool (blast) analysis on the National Center for Biotechnology Information (NCBI) nucleotide database.

### Detection of particulate matter

Particulate matter 2.5 (PM_2.5_) concentrations in the environments were determined using a portable optical particle counter, the SPS30 particulate matter sensor (Sensirion AG, Switzerland). Measurements were performed before operation (at rest) and during operation in the rooms using two independent sensors. PM was measured was measured for 1 h and data collection was repeated on 5 different days.

### Statistical analysis

Means ranks of the bacterial and fungal counts, and PM_2.5_ in the laboratories and within specific workstations of the pilot manufacturing line were compared using the Kruskal–Wallis test. Non-parametric Spearman correlation was used to determine the relationship between microbial counts and PM_2.5_. All statistical analyses were carried out using GraphPad Prism for Windows version 8.0.2 (GraphPad Prism, RRID:SCR_002798). A *P* value *<*0.05 was considered statistically significant.

## Results

The air sampling results in laser-processing environments are presented in Table 1. Median bacterial count, at rest, was <LOD in the three laboratories, although the ranges vary in the clean room (<LOD – 2 c.f.u./plate), the pilot line facility (<LOD – 3 c.f.u./plate) and the standard laser laboratory (<LOD – 4 c.f.u./plate) (Table 2). During operation, there was a higher range of bacterial contamination in the clean room (<LOD – 8 c.f.u./plate) and pilot line facility (<LOD – 5 c.f.u./plate) compared to the standard laser laboratory (<LOD – 2 c.f.u./plate), although the median bacterial count observed in all the laboratories was below the LOD. Of the three laser materials processing laboratories analysed at rest, the pilot line facility had significantly higher (*P*=0.0014) median fungal count (1.0 c.f.u./plate) compared to the clean room (<LOD) (Table 2). There was no significant difference (*P*>0.05) in the fungal count and range among the laboratories during operation. Overall, there was no significant difference in the bacterial and fungal counts at rest and during operation in the three laser materials processing laboratories.

**Table 2.** Microbial counts in different laser materials processing laboratories

(a) Bacteria				
	**At rest**		**During operation**	
	**Median** **(c.f.u./plate)**	**Range** **(c.f.u./plate)**	**Median** **(c.f.u./plate)**	**Range** **(c.f.u./plate)**
**Clean room**	<LOD*	<LOD – 2	<LOD*	<LOD – 8
**Pilot line facility**	<LOD*	<LOD – 3	<LOD*	<LOD – 5
**Standard laser laboratory**	<LOD*	<LOD – 4	<LOD*	<LOD – 2
**(b) Fungi**				
	**At rest**		**During operation**	
	**Median** **(c.f.u./plate)**	**Range** **(c.f.u./plate)**	**Median** **(c.f.u./plate)**	**Range** **(c.f.u./plate)**
**Clean room**	<LOD^†^	<LOD – 1	<LOD^†^	<LOD – 2
**Pilot line facility**	1.0^‡^	<LOD – 2	<LOD^†^	<LOD – 2
**Standard laser laboratory**	<LOD^†‡^	<LOD – 3	<LOD^†^	<LOD – 2

LOD, limit of detection (1 c.f.u./plate).

*Same letter within a column denotes no significant difference (*P*>0.05) between mean ranks from different environments.

†‡ Different letters within a column denotes a significant difference (*P*<0.05) between mean ranks from different environments.

Microbial counts within the workstations of the pilot manufacturing line, located within pilot line facility, were determined. At rest, although the median bacterial count at workstation 2 was 1 c.f.u./plate, there was no significant difference (*P*>0.05) among them (Table 3). During operation, workstation 2 had a significantly higher (*P*=0.0472) median bacterial count (1.0 c.f.u./plate) than workstation 1, but it was not significantly different (*P*>0.05) from that of workstation 3. For fungal count at rest, the median counts in all the workstations were below the LOD (Table 3). Similar to the results from laboratories, there was no significant difference in the bacterial and fungal counts at rest and during operation within the workstations.

**Table 3.** Microbial counts within the pilot manufacturing line workstations

(a) Bacteria				
	**At rest**		**During operation**	
	**Median** **(c.f.u./plate)**	**Range** **(c.f.u./plate)**	**Median** **(c.f.u./plate)**	**Range** **(c.f.u./plate)**
Workstation 1	<LOD^∗^	<LOD – 3	<LOD^∗^	<LOD – 5
Workstation 2	1.0^∗^	<LOD – 2	1.0^†^	<LOD – 4
Workstation 3	<LOD^∗^	<LOD – 2	<LOD^∗†^	<LOD – 3
**(b) Fungi**				
	**At rest**		**During operation**	
	**Median** **(c.f.u./plate)**	**Range** **(c.f.u./plate)**	**Median** **(c.f.u./plate)**	**Range** **(c.f.u./plate)**
Workstation 1	<LOD^‡^	<LOD – 2	<LOD^‡^	<LOD – 1
Workstation 2	1.0^‡^	<LOD – 1	<LOD^‡^	<LOD – 2
Workstation 3	<LOD^‡^	<LOD – 3	<LOD^‡^	<LOD – 3

LOD, limit of detection (1 c.f.u./plate).

∗†‡-Same letter within a column denotes no significant difference (*P*>0.05) between means from different workstations and channels.

**Fig. 1.**
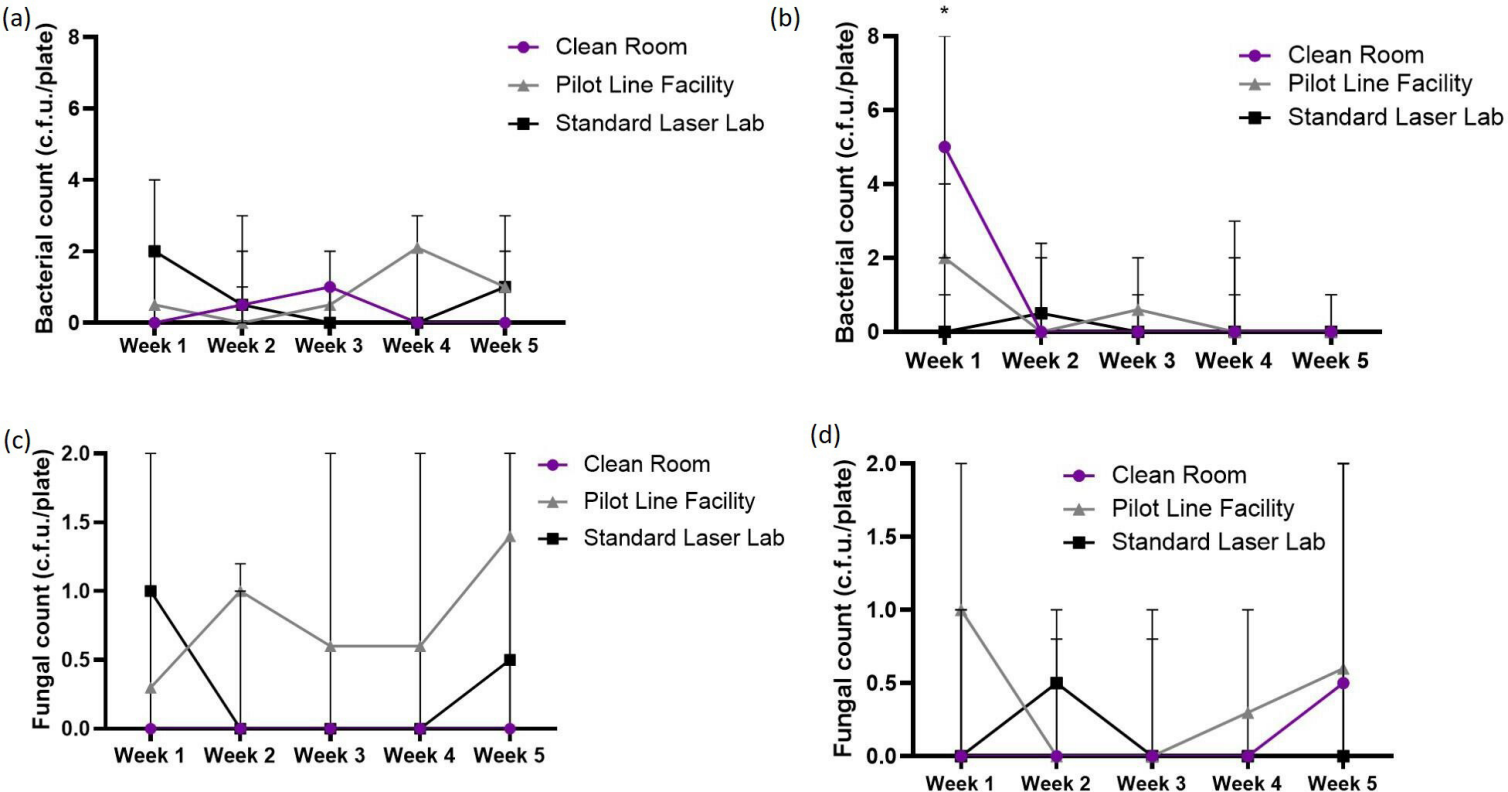
Trend of microbial contaminants in different laser materials processing laboratories. Bacterial count at rest (**a**) and during operation (**b**) as well as fungal count at rest (**c**) and during operation (**d**), with four replicates at each location. Error bars represent 95 % confidence interval (CI) of median.

**Fig. 2.**
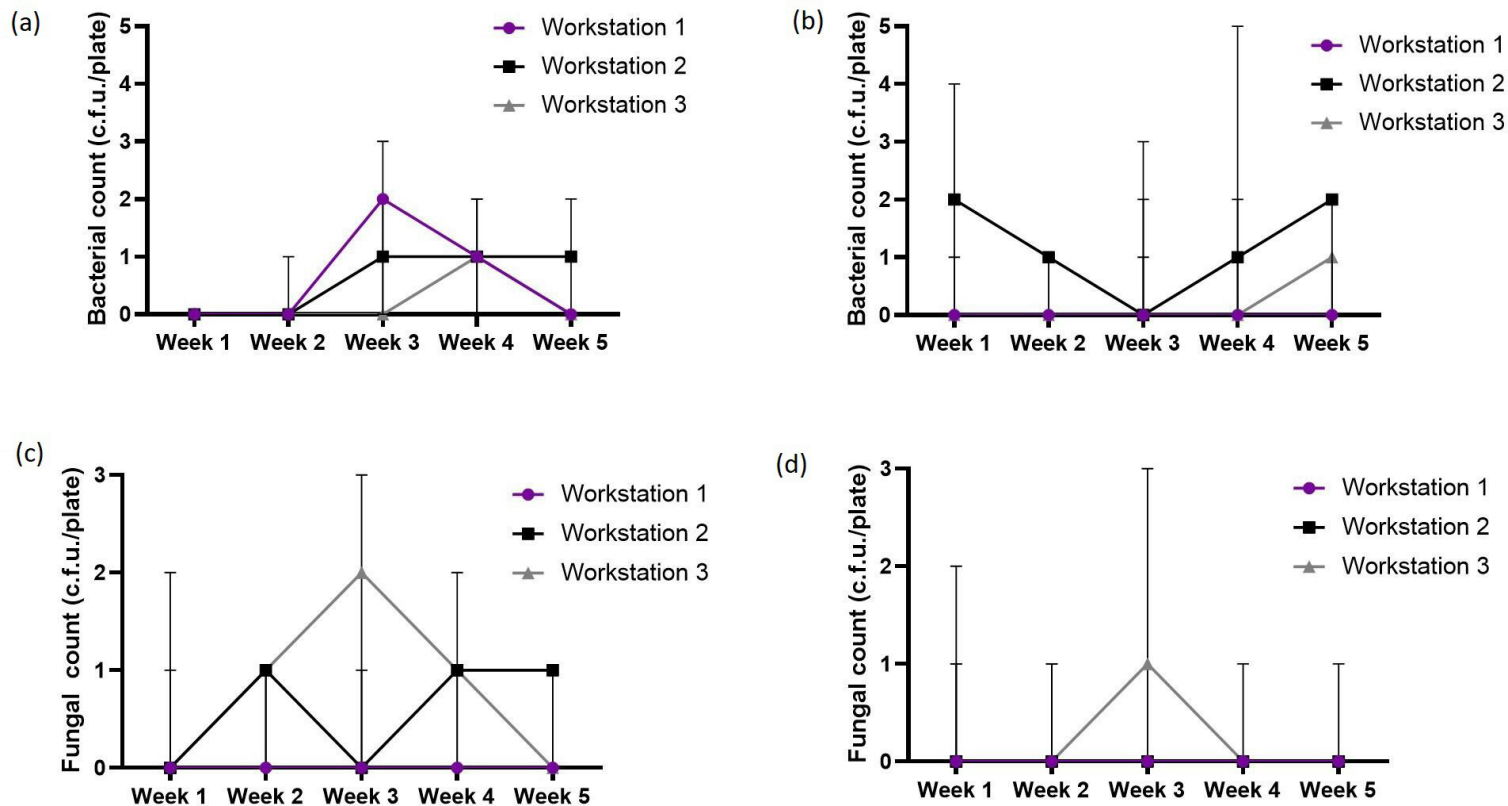
Trend of microbial contaminants within workstations in a pilot manufacturing line: Bacterial count at rest (**a**) and during operation (**b**) as well as fungal count at rest (**c**) and during operation (**d**), with four replicates at each location. Error bars represent 95 % confidence interval (CI) of median.

Data on the monitoring of the bacterial count in the laser materials processing laboratories over 5 weeks are shown in Fig. 1. The observed bacterial counts ranged from <LOD – 8 c.f.u./plate. The bacterial counts during operation were generally lower than at rest, although there was no significant difference (*P*>0.05) between them (Fig. 1a, b). There was no significant difference (*P*>0.05) in the bacterial counts in each location across the weeks except during operation in week 1 (Fig. 1b). During operation in week 1, bacterial count in the clean room (median=5 c.f.u./plate) was significantly higher (*P*=0.021) than in the pilot line facility (median=2 c.f.u./plate) and the standard laser laboratory (median = <LOD). The fungal counts in the laser materials processing laboratories ranged from <LOD – 3 c.f.u./plate. Fungal counts were lower than the bacterial counts both at rest and during operation (Fig. 1c, d). The trend of microbial contamination within the pilot line workstations is shown in Fig. 2. There was no significant difference (*P*>0.05) in the bacterial and fungal counts in each workstation across the weeks (Fig. 2a).

**Fig. 3.**
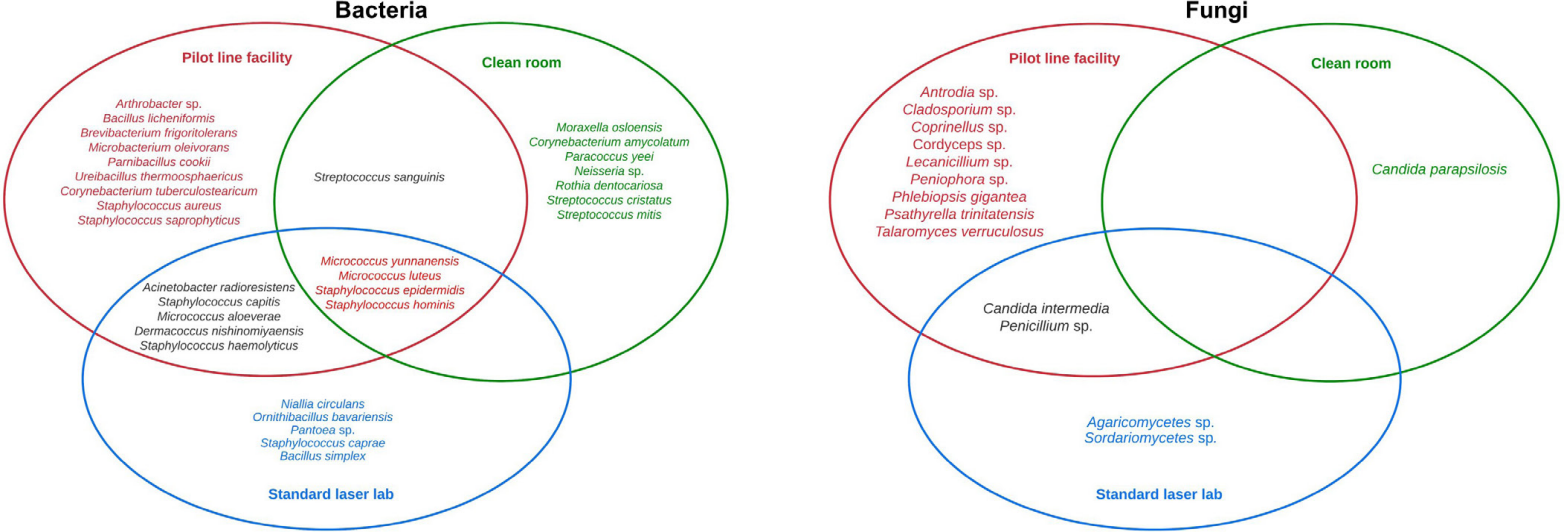
Micro-organisms isolated in different laser materials processing laboratories.

In this study, 20 bacterial genera were isolated in the laser materials processing laboratories (Fig. 3): *Acinetobacter *, *Arthrobacter *, *Bacillus *, *Brachybacterium *, *Brevibacterium *, *Corynebacterium *, *Dermacoccus *, *Microbacterium *, *Micrococcus *, *Moraxella *, *Neisseria *, *Niallia *, *Ornithinibacillus *, *Paenibacillus *, *Pantoea *, *Paracoccus *, *Rothia *, *Staphylococcus *, *Streptococcus* and *Ureibacillus. Staphylococcus* was the most diverse genera, with seven species, namely *Staphylococcus aureus *, *Staphylococcus capitis *, *Staphylococcus caprae *, *Staphylococcus epidermidis *, *Staphylococcus haemolyticus *, *Staphylococcus hominis* and *Staphylococcus saprophyticus. Micrococcus yunnanensis* and *S. epidermidis* were detected in all three laser-processing environments. *Acinetobacter radioresistens* and *Dermacoccus nishinomiyaensis* were isolated in the standard laser and the pilot line facility but not in the clean room. *Paracoccus yeei *, *Neisseria* sp., *Rothia dentocariosa, Streptococcus cristatus* and *Streptococcus mitis* were only detected in clean room. *Niallia circulans, Ornithinibacillus bavariensis, Pantoea* sp. and *S. caprae* were only detected in the standard laser laboratory, while *Arthrobacter* sp.*, Bacillus licheniformis, Brevibacterium frigoritolerans, Microbacterium oleivorans, Paenibacillus cookie* and *Ureibacillus thermosphaericus* were only detected in the pilot line facility.

**Fig. 4.**
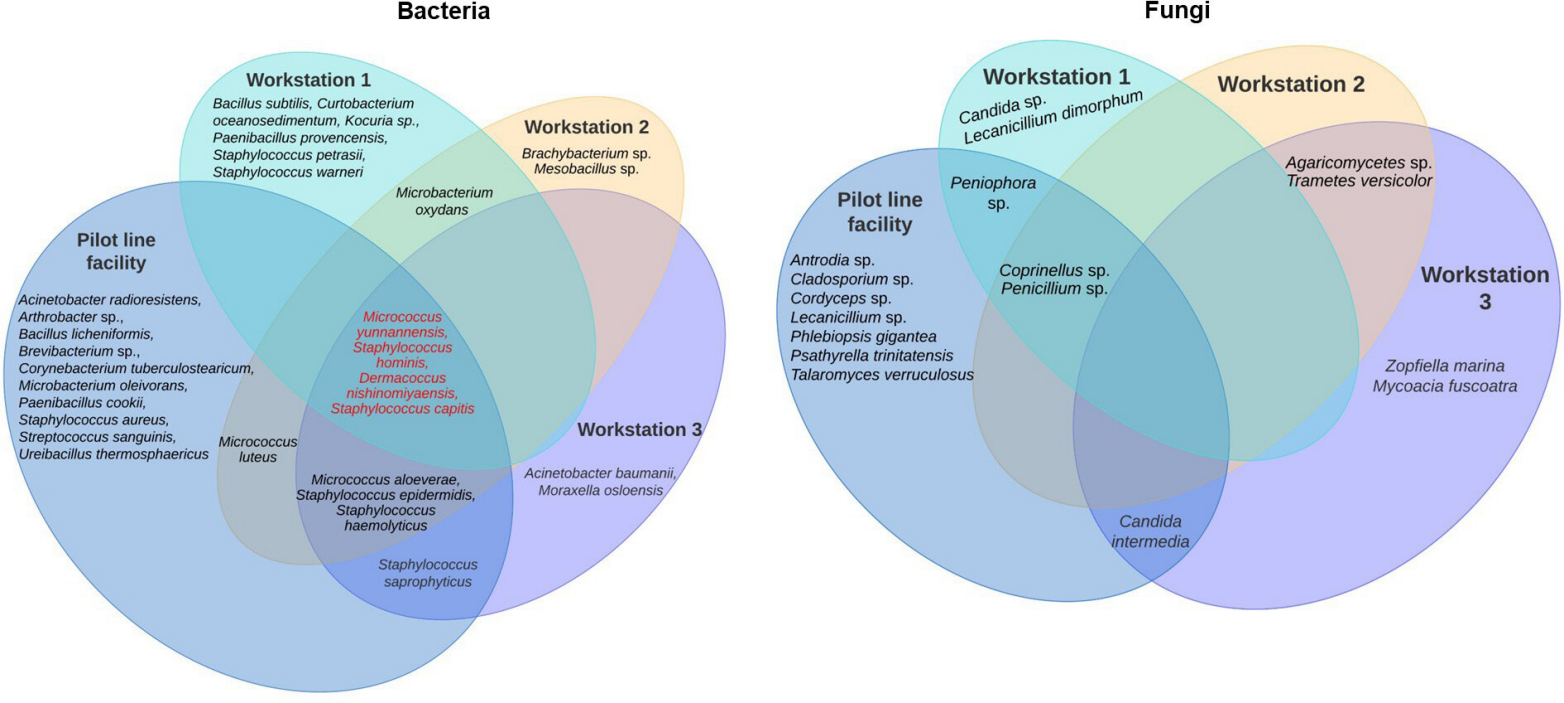
Distribution of isolates in the pilot manufacturing line workstations located within the pilot line facility.

A total of 16 fungal genera were isolated in this study; 13 genera were isolated in the laser materials processing laboratories (Fig. 3) and 9 genera were isolated within the pilot line workstations (Fig. 4). The fungal genera isolated from the laser materials processing laboratories were: *Agaricomycetes*, *Antrodia*, *Candida*, *Cladosporium*, *Coprinellus*, *Cordyceps*, *Lecanicillium*, *Penicillium*, *Peniophora*, *Phlebiopsis*, *Psathyrella*, *Sordariomycetes* and *Talaromyces*. The genera isolated within the workstations were *Agaricomycetes, Candida*, *Coprinellus*, *Lecanicillium*, *Mycoacia*, *Penicillium*, *Peniophora*, *Trametes* and *Zopfiella. Candida parapsilosis* was the only fungus detected in the clean room. *Candida intermedia* and *Penicillium* sp. were only isolated in the standard laser laboratory and the pilot line facility and not in the clean room. *Sordariomycetes* sp. and *Agaricomycetes* sp. were only detected in the standard laser laboratory, while *Coprinellus* sp.*, Cladosporium* sp., *Cordyceps* sp., *Peniophora* sp., *Psathyrella trinitatensis*, *Lecanicillium* sp*.*, *Phlebiopsis gigantea* and *Talaromyces verruculosus* were only detected in the pilot line facility.

The thirteen genera isolated in the pilot line facility were *Acinetobacter *, *Bacillus *, *Brachybacterium *, *Curtobacterium *, *Dermacoccus *, *Kocuria *, *Mesobacillus *, *Microbacterium *, *Micrococcus *, *Moraxella *, *Paenibacillus *, *Paracoccus* and *Staphylococcus. Curtobacterium*, *Kocuria* and *Mesobacillus* were found in the pilot line facility but not in other laser materials processing laboratories. *M. yunnanensis* and *D. nishinomiyaensis* were found in all the workstations. *S. hominis* and *S. capitis* were detected in all workstations. *S. epidermidis* was detected in all workstations. *Microbacterium oxydans* was only found in workstations 1 and 2, whereas *Micrococcus aloeverae* and *S. haemolyticus* were detected in workstations 2 and 3. *Bacillus subtilis *, *Curtobacterium oceanosedimentum, Kocuria* sp.*, Paenibacillus provencensis*, *Staphylococcus petrasii* and *Staphylococcus warneri* were only detected in workstation 1. The isolates found solely in workstation 2 were *Paracoccus yeei, Brachybacterium* sp., *Mesobacillus* and *Micrococcus luteus. A. baumannii*, *Moraxella osloensis* and *S. saprophyticus* were only detected in workstation 3. Isolates detected within the pilot line workstations but not found in the pilot line facility include *A. baumannii, Bacillus simplex, Brachybacterium* sp., *C. oceanosedimentum, Kocuria* sp., *Mesobacillus* sp., *M. oxydans, M. osloensis**,** P. provencensis, P. yeei, S. petrasii* and *S. warneri *.

Fungal isolates from workstation 1 had the least diversity among the pilot line workstations, as there were only three fungal isolates, while workstation 3 had five genera (Table 4). *Peniophora* sp. and *Lecanicillium dimorphum* were only detected in workstation 1; *Coprinellus* sp. and *Penicillium* sp. were only detected in workstation 2, while *Zopfiella marina* and *Mycoacia fuscoatra* were only detected in workstation 3. *Agaricomycetes* sp., *C. intermedia*, *Coprinellus* sp., *Lecanicillium* sp., *Penicillium* sp. and *Peniophora* sp. were detected both within the pilot line workstations and in the pilot line facility. However, *M. fuscoatra*, *Trametes versicolor* and *Z. marina* were found in the workstations but not in the pilot line facility. Furthermore, *C. parapsilosis, Sordariomycetes* sp*., Antrodia* sp*., Cladosporium* sp*., Cordyceps* sp*., P. trinitatensis and T. verruculosus* were found in the pilot line facility but not within the pilot line workstations.

**Table 4.** Concentration of particulate matter (PM_2.5_) in different laser materials processing laboratories

		**Clean room**		**Pilot line facility**		**Standard laser laboratory**	
		**Median**	**Range**	**Median**	**Range**	**Median**	**Range**
**Mass concentration** (**µg m^−3^**)	**At rest**	0.0^∗^	0.0–0.03	1.05^†^	0.96–1.9	0.92^†^	0.66–1.0
	**During operation**	0.03^∗^	0.01–0.08	1.08^†^	0.71–1.90	nd	nd
**Number concentration** (**N m^−3^**)	**At rest**	0^∗^	0–5.9×10^4^	7.8×10^†6^	6.85×10^6^ – 1.43×10^7^	6.24×10^6†^	4.81×10^6^ – 7.22×10^6^
	**During operation**	7.04×10^4∗^	5.71×10^4^ – 1.64×10^5^	7.8×10^†6^	5.13×10^6^ – 1.44×10^7^	nd	nd

nd - not determined.

∗†- Different letters in a row denotes a significant difference (*P*<0.05) between mean ranks from different environments.

To determine if there was a relationship between the concentrations of particulate matter and microbial population in the laser-processing environment, the concentrations of particulate matter (PM_2.5_) at rest and during operation were determined in the three laser-processing environments. Of the three laser-processing environments at rest, the clean room had the significantly lowest (*P*<0.0001) PM_2.5_ mass concentration (median=0.01 µg m^−3^) and number concentration (median=1.29×10^4^ particles m^−3^), while the pilot line facility had the highest median mass concentration (1.33 µg m^−3^) and median mass concentration (9.87×10^6^ particles m^−3^) (Table 4). There was no significant difference (*P*>0.05) between the mass and number concentrations of the pilot line facility and the standard laser laboratory. The standard laser laboratory was not in use during the PM_2.5_ determination, hence no data were collected. Similar to the data obtained at rest, there was a significant difference (*P*<0.0001) between PM_2.5_ mass concentration and number concentration of the pilot line facility and clean room during operation, with the pilot line facility having higher mass- (median=1.22 µg m^−3^) and number concentrations (median=8.99×10^6^ particles m^−3^) than the clean room (median mass concentration=0.03 µg m^−3^; median number concentration=9.24×10^4^ particles m^−3^). Significantly higher PM_2.5_ mass and number concentrations were observed in the clean room during operation than when at rest, unlike in the pilot line facility, where there was no significant difference in the mass and number concentrations at rest and during operation.

There was a weak positive correlation between bacterial count and PM_2.5_ mass concentration (Spearman r=0.09; *P*=0.85) and between bacterial count and PM_2.5_ number concentration (Spearman r=0.14; *P*=0.76). Furthermore, there was a weak negative correlation between fungal count and PM_2.5_ mass concentration (Spearman r=−0.16; *P*=0.75) and between bacterial count and PM_2.5_ number concentration (Spearman r=−0.05; *P*=0.93). Overall, there was no significant correlation between microbial count and PM_2.5_ concentration in the different laser materials processing laboratories.

## Discussion

This study describes microbial contaminants present in laser laboratories and environments involved in the pilot production of biomedical devices. The microbial contamination counts observed in this study were within the recommended limits for the ISO class 7, equivalent to grade C according to the European Union (EU) Good Manufacturing Practice (GMP) [17]. No microbial count at any of the weeks exceeded the maximum acceptable levels of index of microbial air contamination in very high-risk environments (5 c.f.u./plate), such as ultra clean rooms [18]. To our knowledge, this is the first study of its kind in laser laboratories. The bacterial and fungal counts in this study were also compared to those for a single-occupant office environment and there were higher populations in the office (bacteria=7.33 c.f.u./plate; fungi=2.33 c.f.u./plate). Furthermore, the counts observed in the laser-processing environments were lower than those reported in a tissue culture laboratory [19], libraries [20, 21], other indoor environments [22, 23] and food production environments [24–26]. Furthermore, the microbial counts found in this study were lower than those reported in other controlled environments, such as operating theatres (at rest=7.2×10^2^ c.f.u./plate and during operation=1.05×10^4^ c.f.u./plate) [27]. The authors [27] found a strong significant correlation (Spearman’s rank correlation coefficient=0.96–0.99) between active (volumetric) air sampling and passive (settle plate) sampling.

In this study, there was no significant difference between the bacterial counts at rest and during operation in the three laser laboratories. This could be because of the use of PPE, particularly facemasks, by the few personnel working in the laboratories as part of coronavirus disease 2019 (COVID-19) mitigation strategies in the workplace during the sampling period [28]. Wearing of facemasks reduces the dissemination of aerosols and micro-organisms from the mouth, nose and face into the environment [29]. The predominant bacterial genus found in this study was *Staphylococcus *, with seven species. All seven staphylococcal species observed in this study have been previously found in living rooms [30]. *Micrococcus yunnanensis* and *Staphylococcus epidermidis* were detected in all three laser-processing environments and they have been isolated in various indoor environments such as homes, classrooms, research laboratories and hospitals [31, 32]. *Micrococcus* and *Staphylococcus* sp., along with *Corynebacterium* sp., are associated with human skin [33]. Many of the isolates obtained during operation in the clean room, such as *Rothia *, *Neisseria* and *Streptococcus* sp., are associated with the oral cavity and upper respiratory tract [34]. This suggests that face coverings might not have been used during the operation, hence the need for appropriate use of face coverings during operation in the clean room.

*Acinetobacter radioresistens *, a radiation-resistant bacterium found on skin, was only isolated in the uncontrolled environments, i.e. the pilot line facility and standard laser laboratory. This bacterium is a source of opportunistic infections and a major source of carbapenem resistance [35]. Others, such as *Dermacoccus nishinomiyaensis *, *Staphylococcus capitis* and *Staphylococcus haemolyticus *, are also associated with the skin and can cause infections in humans [36–38], hence the need to cover the face and bare skin when working in the laser-processing environments. Most of the isolates unique to both the pilot line facility and standard laser laboratory were predominantly environmental organisms related to the external environment, particularly the soil. This indicates the importance of wearing appropriate PPE, such as shoe coverings, in uncontrolled laser-processing environments in order to reduce microbial contamination. While the micro-organisms in the pilot line workstations shared some similarities with the pilot line facility, in which they are located, there were still some isolates found in the workstations but not in the pilot line facility. For example, *Acinetobacter baumannii* was isolated in workstation 3, which raises a huge health concern, as it is an important pathogen capable of causing multiple infections in humans [39], particularly among the immunocompromised and critically ill patients. Environmental *A. baumannii* isolates have been reported to harbour multiple drug-resistant genes [40] and carbapenem-resistant *A. baumannii* has been identified to be of critical priority for developing new antibiotics [41].

The only fungus isolated in the clean room was *Candida parapsilosis*, which is a commensal of humans but is also isolated in soil. This fungus poses a significant health threat because of its role in invasive infections such as bloodstream infections [42]. Furthermore, *C. parapsilosis* has been identified as an emerging antifungal-resistant organism worldwide [43]. *Penicillium* sp. and *Candida intermedia* were found in the pilot line facility and standard laser laboratory. *Penicillium* sp. is a common fungus found in indoor and outdoor environments, and its indoor levels have been associated with asthma morbidity [44]. On the other hand, *C. intermedia*, which predominantly occurs on human skin and in the throat but also in soil, can cause blood infections in humans [45].

The significantly higher fungal count observed in the pilot line facility in this study could be due to the laboratory’s proximity to the general corridor and the fact that no footwear covering is worn, which may transfer fungi from outdoor environments into the laboratory. It has been reported that fungi in indoor air are dominated by fungi from outdoor air [46]. Conversely, the significantly lower fungal counts found in the clean room could be due to the clean room being further away from the corridor and having a changing room where laboratory coats and shoe coverings are worn prior to entry. Further, the presence of air filtration/ventilation systems, such as HEPA filters, in the clean room may also reduce fungal counts [47]. Continuous ventilation is recommended for reducing finer particulate matter (<30 nm) in indoor environments as it reduces mass concentration of particulate matter faster than when motion sensor-regulated ventilators are used [48]. This point could be further corroborated by the fact that only one fungal isolate was detected in the clean room, which is a controlled environment, compared to the pilot line facility and standard laser laboratory, which are uncontrolled environments. Since some of the fungal isolates in the indoor laser-processing environments, particularly the uncontrolled environments, have also previously been reported in outdoor environments, they may have originated from the outdoor air [49].

The mass concentration of PM_2.5_ (range=0.00–3.76 µg m^−3^) detected in this study were below the EU air quality standard limit of 20 µg m^−3^ for PM_2.5_ [50]. The low particle numbers in this study could be because of the requirement of PPE use based on the COVID-19 pandemic guidelines for working in laboratories. Similar to this study, Landrin *et al*. [51] found no significant correlation between particle counting and microbiological sampling, although correlations between bacterial counts by passive sampling and PM_2.5_ in hospital environments have been reported [52].

In conclusion, this study showed that there is a low level of microbial contamination in laser-processing environments. Twenty bacterial and 16 fungal genera were isolated in this study, with the pilot line facility having the highest microbial diversity (13 bacterial and 11 fungal genera). Most of the isolates are associated with skin, mouth and upper respiratory tract, but some are potentially pathogenic. *Staphylococcus* is the most common and most diverse genus isolated. Finally, there was no significant correlation between the microbial count and PM_2.5_ concentration in the laser-processing environments. These results provide data that will be useful for developing a contamination control plan for controlling microbial contamination and facilitating advanced manufacturing of laser-based pilot production of medical devices. Based on our results, it is recommended that shoe covers and facemasks be worn in laboratories used for prototyping medical devices to minimize microbial contamination.
